# GC/MS-Based Metabolomic Analysis of A549 Cells Exposed to Emerging Organophosphate Flame Retardants

**DOI:** 10.3390/toxics12060384

**Published:** 2024-05-24

**Authors:** Mengyao Sun, Xiao Chang, Ying Gao, Sisi Zou, Shaomin Wang, Hongmin Liu

**Affiliations:** 1 School of Ecology and Environment, Zhengzhou University, Zhengzhou 450001, China; 2 College of Chemistry, Zhengzhou University, Zhengzhou 450052, China; 3 College of Resources and Environment, University of Chinese Academy of Sciences, Beijing 100049, China; 4 School of Pharmaceutical Sciences, Zhengzhou University, Zhengzhou 450001, China

**Keywords:** emerging organophosphate flame retardants, A549 cells, cytotoxicity, gas chromatography/mass spectrometry, metabolomic analysis

## Abstract

Emerging organophosphate flame retardants (eOPFRs) have attracted attention in recent times and are expected to gain extensive usage in the coming years. However, they may have adverse effects on organisms. Due to their novel nature, there are few relevant articles dealing with toxicological studies of the above eOPFRs, especially their information on the perturbation of cellular metabolism, which is, thus far, marginally understood. Our research initially assessed the cytotoxicity of eOPFRs, which include compounds like cresyl diphenyl phosphate (CDP), resorcinol bis(diphenyl phosphate) (RDP), triallyl phosphate (TAP), and pentaerythritol phosphate alcohol (PEPA). This evaluation was conducted using the methyl thiazolyl tetrazolium (MTT) assay. Subsequently, we utilized a gas chromatography/mass spectrometry (GC/MS)-based metabolomic approach to investigate the metabolic disruptions induced by these four eOPFRs in A549 cells. The MTT results showed that, at high concentrations of 1 mM, their cytotoxicity was ranked as CDP > TAP > RDP > PEPA. In addition, metabolic studies at low concentrations of 10 μM showed that the metabolic interference of CDP, TAP, and PEPA focuses on oxidative stress, amino acid metabolism, and energy metabolism, while RDP mainly affects energy metabolism—galactose metabolism and gluconeogenesis. Therefore, from the perspective of cytotoxicity and metabolic analysis, RDP may be a more promising alternative. Our experiments provide important insights into the possible metabolic effects of potential toxic substances and complement the evidence on the human health risks of eOPFRs.

## 1. Introduction

Emerging organophosphate flame retardants (eOPFRs) represent a newly recognized category of organophosphate flame retardants (OPFRs), either recently introduced to the market or recently identified in environmental studies [1,2]. The demand for eOPFRs is set to rise because of the phasing out of or global restrictions imposed on certain brominated flame retardants (BFRs), with some OPFRs facing worldwide bans or limitations [3,4]. eOPFRs are heat-resistant organic compounds containing phosphorus, and they are already incorporated into certain products [5]. Similar to traditional flame retardants, eOPFRs are primarily physically added to products, making it easier for them to enter the ambient medium [6]. These contaminants have the potential to pose a threat to human health by entering the body through the unintentional ingestion from hand-to-mouth contact, absorption through the skin, or inhalation of suspended dust particles [7,8]. The eOPFRs examined in this study include cresyl diphenyl phosphate (CDP), resorcinol bis(diphenyl phosphate) (RDP), triallyl phosphate (TAP), and pentaerythritol phosphate alcohol (PEPA).

CDP has a wide range of applications and can be used as a plasticizer or a flame retardant [4]. It has been identified in indoor dust across various countries, with maximum concentrations of 38.9 μg/g dry weight [9]. Studies have shown that CDP can damage DNA and mitochondria and can lead to cell-cycle arrest [10]. It also has reproductive and developmental toxicity [11], cardiac developmental toxicity [12], and endocrine disrupting properties [13]. RDP is mainly used to replace triphenyl phosphate, tris (2-chloroethyl) phosphate, and tris (1,3-dichloroisoprophyl) phosphate [14,15]. This is because RDP is less volatile, has a higher thermal stability, and contains more phosphorus than triphenyl phosphate [16]. It was reported that RDP is present in fumes and aerosols released during its production [4]. The bioconcentration factor (BCF) for the RDP was 15,546.47 [17], suggesting that some bioaccumulation may occur. Hou et al. noted that RDP concentrations ranged from <MDL to 0.370 ng/mL in whole blood samples from 76 elderly people in Jinan, China [18]. TAP can be added to unsaturated polyester resin and high voltage lithium-ion cell electrolytes [19,20] to play the dual role of flame retardant and improve the product performance. It is speculated that, in the future, the dosage of TAP will increase, but there is no research on the biological toxicity of TAP at present. PEPA, with a caged bicyclic structure, presents a high flame retardant efficiency. At present, an increasing number of studies have been conducted to synthesize new epoxy/PEPA phosphate flame retardants based on PEPA [21,22,23]. Notably, there are currently no studies reported on the toxicology of PEPA.

Metabolomics has the ability to qualify and quantify metabolites in cells [24]. By generating a profile of small molecules that are derived from cellular metabolism, metabolomics can directly reflect the outcome of complex networks of biochemical reactions, providing insights into multiple aspects of cellular physiology [25]. The metabolomics analysis of cells has many potential advantages over other methods currently used to study cellular functions [26]. In recent years, the scientific community has increasingly applied metabolomics to solve key multifaceted questions—environmental toxicology is one of them [24]. Metabolomics has demonstrated its superiority in detecting metabolic changes in organisms or cells caused by potential pollutants, even when there are no obvious differences in traditional cognitive phenotypes such as morphology, mortality, reproduction, and weight loss. This allows us to identify the health risks of these potential pollutants at the metabolic level [27]. In our research, to identify relatively more metabolites, gas chromatography/mass spectrometry (GC/MS) was used for the metabolomic analysis of cell extracts. GC/MS has excellent robustness, reproducibility, selectivity, and well-established metabolite databases and has been recognized as one of the most widely applied analytical platforms for metabolomics studies [28,29].

The human lung cancer cell line A549 is a widely used adenocarcinoma cell line that is sensitive to various stimuli and can be used as an in vitro model for respiratory toxicology studies [30,31]. Therefore, A549 cell line was selected as an in vitro test model. In the present work, the cytotoxicities of CDP, RDP, TAP, and PEPA, and their effects on the metabolic networks of the A549 cell line were investigated. The aim of the present study was to investigate the potential effects of eOPFRs on metabolism using cellular models with three components: (I) evaluate the cytotoxicity of eOPFRs; (II) provide data for the exposure-induced respiratory toxicity and related mechanisms of these compounds at the metabolic level; and (III) provide evidence of the health risk of eOPFRs for humans.

## 2. Materials and Methods

### 2.1. Chemicals and Reagents

CDP (93%) was sourced from J&K Scientific (Beijing, China). RDP (98%) and 2-aminobutyric acid (internal standard, 99%) were acquired from Macklin (Shanghai, China). TAP (98%) was obtained from Bidepharm (Shanghai, China). PEPA (98%) was purchased from TCI (Shanghai, China). The comprehensive information of the above chemicals is tabulated in Table 1. These chemicals were dissolved in dimethyl sulfoxide (DMSO, 99%) gained from Solarbio (Beijing, China), at a concentration of 1 M and stored at 4 °C, respectively. Prior to using, the chemical stock solution was diluted with complete medium containing 89% Dulbecco’s Modified Eagle Medium (DMEM)/high-glucose medium (HyClone, Logan, UT, USA), 10% fetal bovine serum (Cell-Box, Hong Kong, China), and 1% Penicillin-Streptomycin solution (Beyotime, Shanghai, China). The final concentration of DMSO in the medium should be less than 1 ‰ (*v*/*v*).

All of the compounds (carbohydrates, fatty acids, and amino acids) and solvents needed for the preparation of standard mixtures were analytical-grade or higher quality and sourced from J&K Scientific (Beijing, China). Methanol (MeOH, HPLC grade) used for metabolite extraction was purchased from Merck (Darmstadt, Germany). Pyridine and methoxylamine hydrochloride used for the preparation of oxime reagents were obtained from Sigma Aldrich (St. Louis, MO, USA). Methyl thiazolyl tetrazolium (MTT, 98%) and silylating reagent trimethylsilyl cyanide (TMSCN, 98.5%) were purchased from HEOWNS (Tianjin, China). Ultra-pure water was obtained from a Milli-Q system (Millipore, MA, USA).

### 2.2. Cell Culture

A549 cells were provided by the School of Pharmaceutical Sciences, Zhengzhou University and cultured in our laboratory. The cells were cultured in a Petri dish, 6 cm in diameter, in 4 mL of complete medium. These cells were cultured with 5% CO_2_ (*v*/*v*) at 37 °C and kept moist. All experiments required passaging cells every 3 days for seeding during the exponential growth phase.

### 2.3. MTT Cell Viability Assay

Using the MTT colorimetry method, the effects of these four eOPFRs on the viability of A549 cells were evaluated. First, A549 cells were collected from a Petri dish, seeded in 96-well plates (JET BIOFIL; Guangzhou, China) with 100 μL of complete medium, and cultured for approximately 24 h (5000 cells/well). Next, these cells were exposed to various concentrations (0.1, 1, 10, 100, and 1000 μM) of the four eOPFRs in fresh culture medium for 48 h. Every eOPFR was dissolved in DMSO first and then diluted with complete medium to reach a final concentration of DMSO at 1 ‰ (*v*/*v*); controls were given the same course of action. After 48 h, MTT solution (0.5 mg/mL) was added to each well, and then the wells were incubated for 4 h. After that, the absorbances were measured at 570 nm using a microplate reader (LabServ K3 Touch, Thermo Fisher Scientific, Waltham, MA, USA). Cell viability was calculated as follows:

$$Cell\;viability(\%)=\frac{{OD}_{test}-{OD}_{kb}}{{OD}_{control}-{OD}_{kb}}$$

Note: test groups (cells + complete medium + OPFR); control groups (cells + complete medium); kb groups (complete medium).

### 2.4. Cell Sample Pretreatment for Metabolomic Analysis

Based on the viability assay results, A549 cells were treated with 10 μM eOPFRs for 48 h for metabolomic analysis. After 48 h of cell culture, the medium was removed from each well. Subsequently, the cells were washed three times with saline at 4 °C, and then immediately quenched in an ultralow temperature refrigerator at −80 °C to arrest cellular metabolic activity. Next, a cell scraper was used to collect cells, and then the cells were lysed through three freezing–thawing cycles (i.e., 1 h at −80 °C, followed by 0.5 h at 37 °C) to increase the extraction efficiency of intracellular metabolites.

Metabolite extraction: After vortexing vigorously for 60 s, 90 μL of internal standard 2-aminobutyric acid and 600 μL of extraction reagent methyl tertiary butyl ether-methanol (1:9, *v*/*v*) were added to cell samples, and then each sample was extracted at 4 °C for 60 min. Precipitated proteins in samples were separated by centrifugation at 12,000× *g* for 10 min in a high-speed freezing (4 °C) centrifuge. After that, the supernatant of each sample was collected and transferred to a separate derivation flask, and then evaporated to dryness in nitrogen at 37 °C to remove organic solvents and water.

Metabolite derivatization: In our experiments, we employed a two-step derivatization method by methoxyamination and silylation. In the first step, 30 μL of a 10 mg/mL pyridine solution of methoxyamine hydrochloride was added to the nitrogen-blown extract, mixed by vortexing, and then allowed to stand for an hour at 70 °C. In the second step, 60 μL of TMSCN was added to each methoximation sample and placed at 50 °C for 1 h [32].

Finally, the processed samples were analyzed by GC/MS. (Instrument information and parameter settings can be found in Supplementary Materials).

### 2.5. Metabolic Data Processing and Multivariate Statistical Analysis

The raw chromatograms were subjected to peak deconvolution using Automated Mass Spectral Deconvolution and Identification Software (AMDIS), the software is freely available on www.amdis.net. Subsequently, metabolites were identified by referencing the National Institute of Standards and Technology (NIST) database and standard substances. The identified metabolites are shown in Table S1 with the chromatogram of GC/MS shown in Figure S1. The relative concentration was obtained by normalizing the peak area of the characteristic ion of the metabolite to the area of the internal standard in the sample. SIMCA-P14.1 software (Umetrics AB, Umea, Sweden) was used for unsupervised and supervised data analysis. The orthogonal projections to latent structures–discriminant analysis (OPLS-DA) model was used to reduce the data dimensionality, obtain the classification and aggregation information of samples, and analyze the VIP values of metabolites. The independent-sample t-test was selected to conduct difference analysis by SPSS 22.0 software. The reliability of the OPLS-DA model was assessed through response permutation testing (RPT).

## 3. Results

### 3.1. Cytotoxicity Analysis of eOPFRs

**Figure 1:**
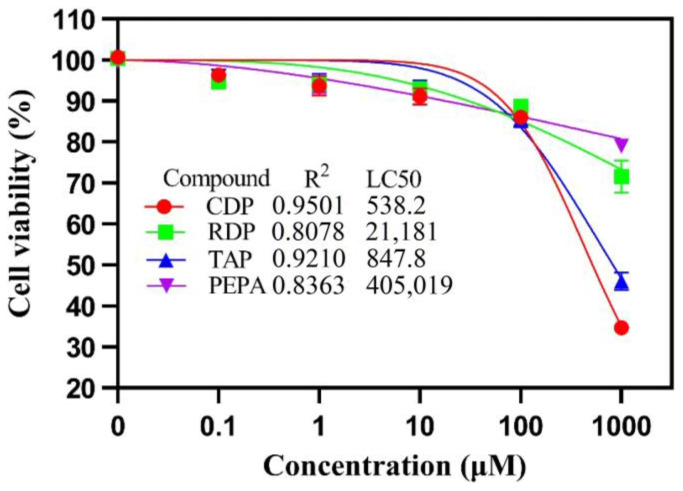
The dose-response curves revealed the cytotoxic effects of eOPFRs on A549 cells, which were fitted using a nonlinear model (inhibitor vs. normalized response—variable slope). The cell viability of A549 cells was evaluated using the MTT assay, with the results expressed as the mean ± SEM (n ≥ 3).

Under the same conditions, the viability of A549 cells was investigated by exposing them to four different flame retardants (CDP, RDP, TAP, and PEPA) in a concentration range of 0 to 1000 μM. As shown in Figure 1, a nonlinear model was employed to fit the scatterplots, with the half-maximal inhibitory concentrations (LC50) of all flame retardants being computed as a result, shown in Figure 1. Meanwhile, a statistical analysis of the data revealed that these eOPFRs displayed a statistically significant (*p* < 0.05) and highly significant (*p* < 0.01) cytotoxicity at the 100 μM and 1000 μM concentrations (Table S2). To preclude the changes in metabolites caused by the reduction in cell viability, we selected 10 μM for the GC/MS analysis under nonsignificant cytotoxic conditions (cell viability greater than 80%).

### 3.2. Metabolomics Analysis

**Figure 2:**
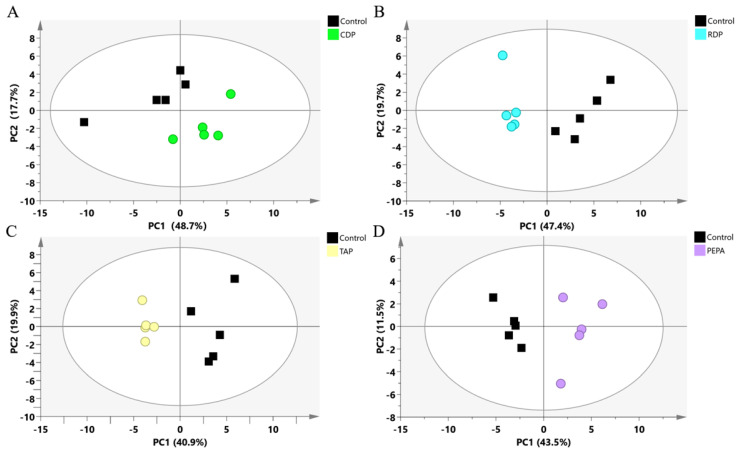
PCA of features derived from GC/MS metabolic profile in different eOPFR treatments: (**A**) solvent control group vs. CDP treatment group (R^2^X (cum) = 0.664, Q^2^ (cum) = 0.260); (**B**) solvent control group vs. RDP treatment group (R^2^X (cum) = 0.671, Q^2^ (cum) = 0.217); (**C**) solvent control group vs. TAP treatment group (R^2^X (cum) = 0.609, Q^2^ (cum) = 0.153); and (**D**) solvent control group vs. PEPA treatment group (R^2^X (cum) = 0.551, Q^2^ (cum) = 0.123). Note: All of the above PCA scores plots have 2 components.

GC/MS was used to conduct a nontargeted metabolomic analysis of cells from a single eOPFR treatment. Metabolic disturbances induced by eOPFR treatment were initially assessed by an unsupervised PCA model using SIMCA software (Figure 2). PCA is an unbiased multivariate statistical method for viewing trends in grouping. The four eOPFRs showed a clear separation from the solvent control group in PCA score plots when visualized (Figure 2).

**Figure 3:**
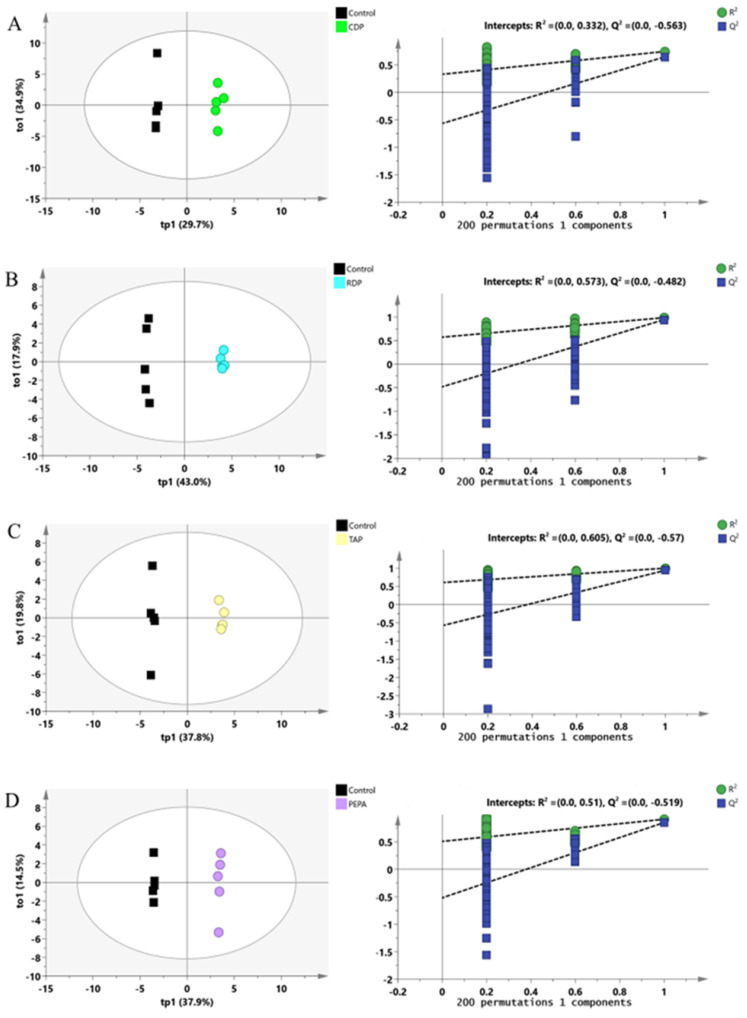
OPLS-DA score plots of GC/MS data derived from the spectra of the cell sample in different eOPFR treatments and validation models for the supervised models generated from cell samples. (**A**) OPLS-DA scores plot for control and CDP-treated group (1 orthogonal and 1 predictive components, R^2^X (cum) = 0.660, R^2^Y (cum) = 0.954, Q^2^ (cum) = 0.831); (**B**) OPLS-DA scores plot for control and RDP-treated groups (1 orthogonal and 1 predictive components, R^2^X (cum) = 0.614, R^2^Y (cum) = 0.988, Q^2^ (cum) = 0.945); (**C**) OPLS-DA scores plot for control and TAP-treated groups (1 orthogonal and 1 predictive components, R^2^X (cum) = 0.583, R^2^Y (cum) = 0.995, Q^2^ (cum) = 0.934); and (**D**) OPLS-DA scores plot for control and PEPA-treated groups (1 orthogonal and 1 predictive components, R^2^X (cum) = 0.530, R^2^Y (cum) = 0.994, Q^2^ (cum) = 0.908).

Hereafter, we employed a supervised multivariate analysis to explore the differentially expressed metabolites (DEMs) that distinguish the eOPFR-treated group from the solvent control group. The OPLS-DA model was employed to narrow intraclass differences, focusing on between-group changes. The OPLS-DA analysis allowed the identification of variables that contributed significantly to the classification of the sample, which differed significantly between groups. From the OPLS-DA score plots, there were significant differences between the metabolic datasets of cells in the four flame retardant groups and the control group (Figure 3). Simultaneously, a permutation test within the model was performed (Figure 3), thus confirming the robustness of the model.

### 3.3. The Identification of DEMs

**Figure 4:**
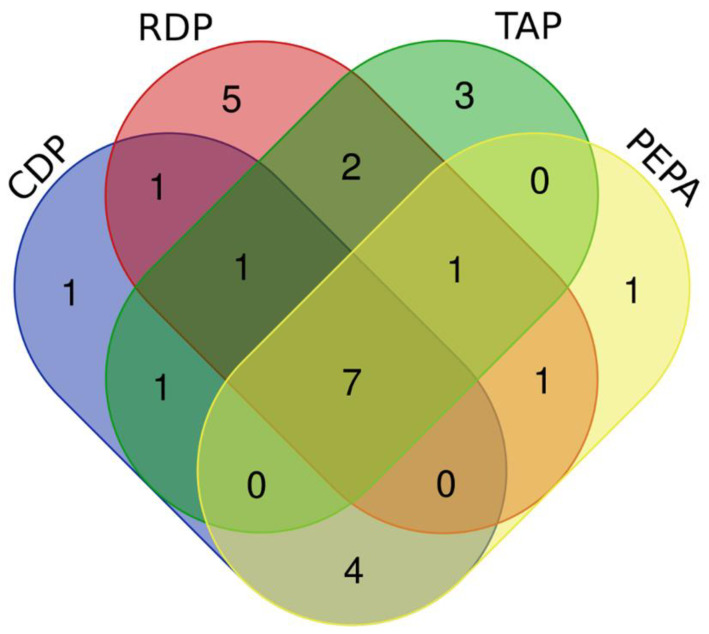
Venn diagram of potential biomarkers of eOPFRs acting on A549 cells.

According to the OPLS-DA model, the data in each group were shown to be completely independent, representing significant metabolic differences due to flame retardant exposure. Metabolites with VIP values > 1 and *p* values < 0.05 were considered significant DEMs [33]. Following CDP exposure, there were 15 obvious DEMs: increased concentrations of glutamic acid and 4-methylcatechol and decreased concentrations of leucine, isoleucine, valine, threonine, glycine, proline, alanine, serine, pyroglutamic acid, lactic acid, gluconic acid, 2-aminomalonic acid, and glucose (Table S3). Following RDP exposure, there were 18 obvious DEMs: valine, threonine, fructose, inositol, isoleucine, mannitol, glucose, erythritol, gluconic acid, leucine, pyroglutamic acid, hexapyranose, proline, malic acid, oleic acid, 2,3-butanediol, 2-aminomalonic acid, and p-hydroxyphenylacetic acid (except for oleic acid and 2,3-butanediol, the concentrations of all other substances decreased) (Table S4). Following TAP exposure, there were 15 obvious DEMs: increased concentrations of fructose, cystathionine, proline, glycol, 4-methylcatechol and erythritol and decreased concentrations of hexapyranose, gluconic acid, glucose, isoleucine, valine, leucine, phenylalanine, threonine, and pyroglutamic acid (Table S5). Following PEPA exposure, there were 14 obvious DEMs: threonine, leucine, valine, isoleucine, alanine, hexapyranose, glucose, gluconic acid, glycine, inositol, serine, pyroglutamic acid, lactic acid, and glyceryl monostearate with downregulated concentrations (Table S6). Notably, the four eOPFRs have seven shared DEMs, including five amino acids (threonine, leucine, valine, isoleucine, and pyroglutamic acid), gluconic acid, and glucose (Figure 4).

### 3.4. Disturbed Pathway Analysis

**Figure 5:**
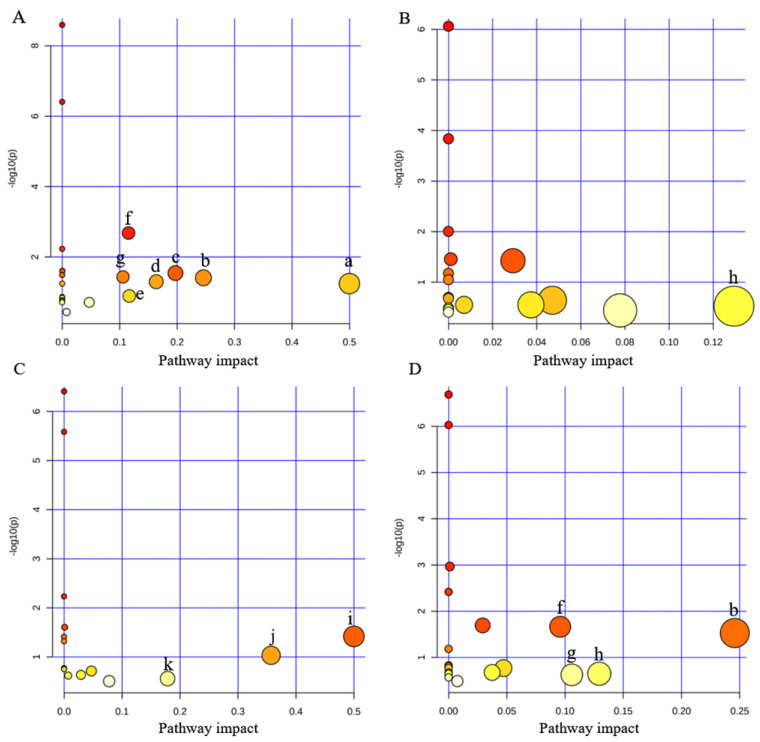
Summary plot of metabolic pathway analysis of eOPFRs treatments performed on A549 cells: (**A**) control group vs. CDP treatments; (**B**) control group vs. RDP treatments; (**C**) control group vs. TAP treatments; and (**D**) control group vs. PEPA treatments. (a) D-glutamine and D-glutamate metabolism; (b) glycine, serine, and threonine metabolism; (c) alanine, aspartate, and glutamate metabolism; (d) arginine and proline metabolism; (e) arginine biosynthesis; (f) glutathione metabolism; (g) glyoxylate and dicarboxylate metabolism; (h) inositol phosphate metabolism; (i) phenylalanine, tyrosine, and tryptophan biosynthesis; (j) phenylalanine metabolism; and (k) cysteine and methionine metabolism.

The pathway analysis was carried out using MetaboAnalyst 5.0 online platform (https://www.metaboanalyst.ca/, accessed on 20 August 2023). This analysis encompassed all pathways that were identified based on the *p*-values obtained from the pathway enrichment analysis and the pathway impact values derived from the pathway topology analysis. The homo sapiens KEGG database was used in this analysis. Metabolic pathways with influence values greater than 0.1 were used as the main metabolic pathways to analyze the potential action mechanism. There were seven main metabolic pathways found (D-glutamine and D-glutamate metabolism; glycine, serine, and threonine metabolism; alanine, aspartate, and glutamate metabolism; arginine and proline metabolism; arginine biosynthesis; glutathione metabolism; and glyoxylate and dicarboxylate metabolism) associated with 15 DEMs identified in CDP exposure (Figure 5A). The inositol phosphate metabolism pathway was disturbed by RDP exposure (Figure 5B). Three key metabolic pathways (phenylalanine, tyrosine, and tryptophan biosynthesis; phenylalanine metabolism; and cysteine and methionine metabolism) were affected under TAP exposure (Figure 5C). PEPA exposure disrupted four metabolic pathways (glycine, serine, and threonine metabolism; glutathione metabolism; glyoxylate and dicarboxylate metabolism; and inositol phosphate metabolism) (Figure 5D).

### 3.5. Metabolite Enrichment Analysis

**Figure 6:**
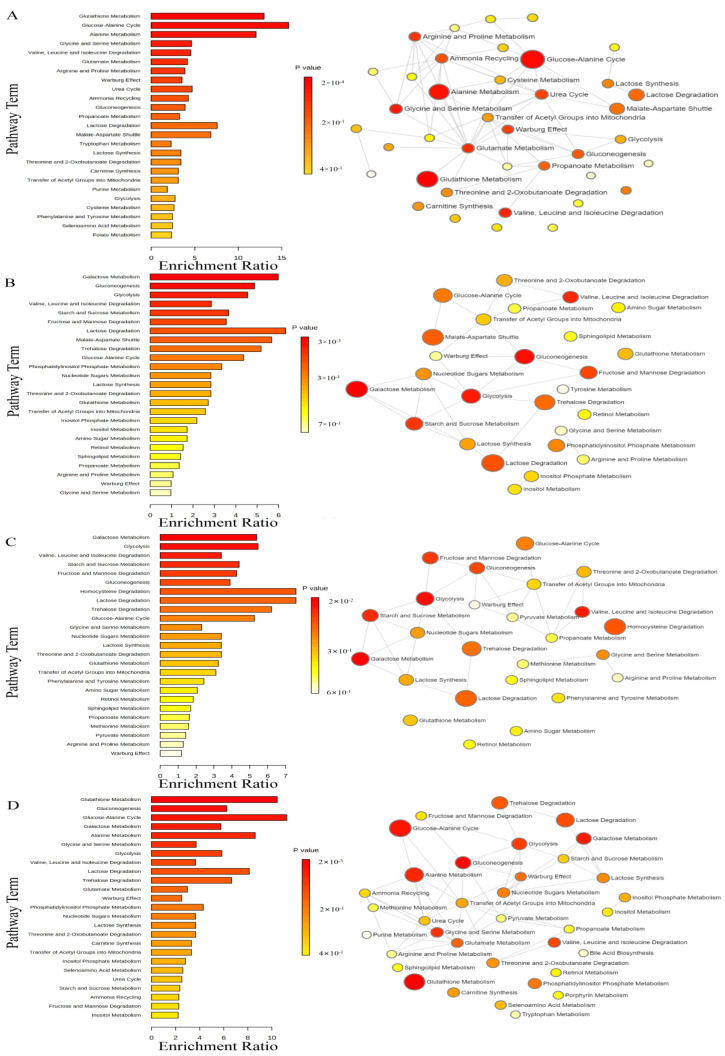
Summary plots of metabolite enrichment analysis of eOPFRs treatments performed on A549 cells (bar charts and network graphs): (**A**) control group vs. CDP treatments; (**B**) control group vs. RDP treatments; (**C**) control group vs. TAP treatments; and (**D**) control group vs. PEPA treatments.

By conducting an enrichment analysis of the DEMs using the SMPDB database (99 metabolite sets based on normal human metabolic pathways), we have determined the possible biochemical processes affected by exposure to eOPFRs and highlighted the correlations between the metabolic pathways. After CDP exposure, statistical significance was found in eight pathways (*p* < 0.05), and the significant biological processes were glutathione metabolism, the glucose–alanine cycle, alanine metabolism, glycine and serine metabolism, valine, leucine, and isoleucine degradation, glutamate metabolism, arginine and proline metabolism, and the Warburg effect (Figure 6A). After RDP exposure, galactose metabolism and gluconeogenesis were significantly different (*p* < 0.05) (Figure 6B). After TAP exposure, the galactose metabolism and glycolysis pathways were significantly different (*p* < 0.05) (Figure 6C). After PEPA exposure, eight pathways were significantly different, including glutathione metabolism, gluconeogenesis, the glucose–alanine cycle, galactose metabolism, alanine metabolism, glycine and serine metabolism, glycolysis, and valine, leucine, and isoleucine degradation (*p* < 0.05) (Figure 6D).

These metabolic pathways are attributed to several essential biological processes, including amino acid metabolism, antioxidant metabolism, and energy metabolism. The metabolic pathways associated with these compounds offer valuable insights into the potential respiratory impairment that may be triggered by eOPFRs.

## 4. Discussion

### 4.1. Cytotoxicity Analysis of eOPFRs Exposure

The effects of flame retardants on the viability of A549 cells were confirmed by treating cells with different concentrations of eOPFR solutions for 48 h. The results showed that all four eOPFRs reduced the viability of A549 cells to different extents in a concentration-dependent manner. As illustrated in Figure 1, when the concentration of four eOPFRs surpassed 10 μM, the cell viability began to show an obvious downwards trend; when the concentration reached 1000 μM, the cell viability dropped sharply, and the cytotoxicity effects were CDP > TAP > RDP > PEPA. The cytotoxic effects of CDP were similar to those in previous studies [10,11].

### 4.2. Analysis of eOPFR-Induced Metabolic Disorders

#### 4.2.1. CDP Induces Oxidative Stress and Disrupts Amino Acid and Energy Metabolism

Compared to the control group, the concentrations of 4-methylcatechol and glutamate increased in the CDP-treated group. In vitro, 4-methylcatechol can induce oxidative stress, which promotes apoptosis in metastatic melanoma cells and murine tumor cells [34,35]. Glutamate, an important amino acid in the human body, is the most abundant neurotransmitter in the mammalian nervous system and has a pro-oxidative effect. Excessive concentrations of glutamate can impede the cellular uptake of cystine through the cystine–glutamate antiporter. This disruption leads to reduced levels of glutathione (GSH) and the buildup of reactive oxygen species (ROS) [36]. GSH is a tripeptide consisting of glutamic acid, glycine, and cysteine. It serves as a vital component in cellular defense mechanisms, acting as both a scavenger of free radicals and a detoxifier to protect against oxidative damage [37]. In the present study, the concentration of glycine (a precursor for the synthesis of GSH) decreased, suggesting that the cell’s antioxidant capacity had changed. Meanwhile, in the CDP-treated group, the concentration of pyroglutamate (a cyclized derivative of glutamate and a metabolite of the GSH cycle) declined [38], which may also suppress the GSH cycle and aggravate the oxidative damage to cells. It is also noteworthy that glutathione metabolism and the D-glutamine and D-glutamic acid metabolic pathways were disrupted in our study, as evidenced in Figure 5A. Glutathione metabolism mainly plays a role in maintaining cell redox homeostasis [39], and the metabolic process of D-glutamine and D-glutamate has a protective antioxidant role [40]. The change in these metabolic pathways also implies an imbalance in the redox state of the cells. Therefore, CDP may damage lung cancer cells by inducing oxidative stress.

According to the pathway analysis and enrichment analysis, four amino acid metabolic pathways were disrupted in the CDP-treated group: glycine, serine, and threonine metabolism; valine, leucine, and isoleucine degradation; arginine and proline metabolism; and arginine biosynthesis (Figure 5A and Figure 6A). These metabolic pathways are linked together through amino acid interactions. Glycine metabolism is interconnected with serine metabolism through enzymes like serine hydroxymethyltransferase and shares transporters with serine and threonine [41]. While serine can be synthesized via the de novo pathway, approximately 20% of serine is generated from glycine due to the activity of serine hydroxymethyltransferase enzymes [42,43]. Threonine is an indispensable amino acid in the human body, and shares biochemical and membrane transport pathways with glycine and serine. Additionally, it plays a role in enhancing cellular immune defense functions [44]. Moreover, glycine, serine, and threonine serve as crucial precursors for the synthesis of proteins, nucleic acids, and lipids [45]. In our study, the concentrations of glycine, serine, and threonine all decreased, which may lead to a reduced immune function of the cells and affect the corresponding protein synthesis in the cells. Valine, leucine, and isoleucine are categorized as branched-chain amino acids (BCAAs), and they play integral roles in the metabolism of blood sugar, fat, and energy [46]. In a study conducted by Yu et al. [47], it was observed that reducing the dietary intake of branched-chain amino acids (BCAAs) in mice can have a positive impact on their metabolic health. Additionally, the research indicated that elevated levels of dietary isoleucine in humans are linked to an increased body mass index (BMI). A diet low in isoleucine has the potential to enhance liver and adipose tissue metabolism by boosting hepatic insulin sensitivity and ketogenesis. Moreover, it may lead to increased energy expenditure and the activation of the FGF21-UCP1 axis. Reducing valine intake can induce comparable but relatively milder metabolic effects. In the experiment, the concentration of BCAAs in the CDP-treated group decreased, suggesting that CDP may affect blood glucose regulation. Proline helps to maintain the equilibrium of cellular conditions and prevent dehydration [48]. Decreased concentrations of proline and increased concentrations of glutamate jointly affect two metabolic pathways, arginine and proline metabolism and arginine biosynthesis, which may lead to a decreased stress resistance in A549 cells.

The glucose–alanine cycle and Warburg effect are the main pathways related to energy metabolism (Figure 6A). The glucose–alanine cycle demonstrates a vital connection between the metabolism of carbohydrates and amino acids [49]. In a recent investigation, it was revealed that, in situations of nutrient scarcity, cancer cells can utilize alanine as an alternative energy source. This utilization triggers metabolic reprogramming in the cancer cells by activating the subsequent glucose–alanine cycle, thereby facilitating cancer cell growth [50]. In addition, under sufficient oxygen and complete mitochondrial function conditions, cancer cells can also obtain energy through a glycolysis pathway known as the Warburg effect or aerobic glycolysis [51,52]. In this study, the large reduction in glucose levels in the treatment group indicated a higher glucose metabolism rate and faster glucose consumption via aerobic glycolysis. At the same time, the alanine concentration decreased slightly, possibly because alanine was used as an alternative energy source to support cancer cell proliferation in the face of excessive glucose consumption.

Taken together, the metabolic disorder between these amino acids and carbohydrates is interconnected. The concentrations of amino acids (except glutamate) and carbohydrates in cells showed various degrees of decline, suggesting that the growth state of A549 cells was affected to some extent, which is likely to be manifested in blocked protein synthesis and energy and lipid metabolism disorders.

#### 4.2.2. Metabolic Pathway Analysis of the Main Effects of RDP

Both RDP and CDP are aryl-OPFRs. In our study, the cytotoxicity and metabolic interference of RDP were weaker than those of CDP. The metabolic pathways disrupted in the RDP treatment group were mainly galactose metabolism and gluconeogenesis (Figure 6B). The DEMs related to galactose metabolism were glucose, fructose, hexapyranose, and inositol. Inositol has an antioxidant function and can repair mitochondria [53]. Carbohydrate metabolism is generally closely related to energy supply and blood sugar regulation. Galactose, an essential source of energy, plays an established role in the energy delivery and galactosylation of intricate molecules. Galactose metabolism is crucial for the human body [54]. The DEMs related to gluconeogenesis were glucose, hexapyranose, and malate. In tumor cells or some immune cells, gluconeogenesis appears to be a significant alternative metabolic pathway to adapt to the microenvironment of glucose scarcity [55]. In the gluconeogenesis pathway, oxaloacetate can be exported from mitochondria to the cytoplasm via the malate shuttle [56]. Moreover, the gluconeogenesis pathway appears to be increased in cancers arising in nongluconeogenic tissues, and downregulating the pathway can inhibit the growth of these tumors (melanoma excepting) [57]. Therefore, we speculated that the reduction in the above metabolites in A549 cells indicates that the galactose metabolism and gluconeogenesis pathways were inhibited, which affected the energy metabolism in cells and induced cell growth inhibition.

#### 4.2.3. TAP-Induced Peculiar Amino Acid Metabolism Disorders

This study observed that TAP led to a reduction in cellular phenylalanine levels, consequently affecting the biosynthesis of phenylalanine, tyrosine, and tryptophan, along with phenylalanine metabolism (Figure 5C). It is important to note that both phenylalanine and tryptophan are amino acids essential for the body, and tyrosine can be synthesized from phenylalanine through the enzymatic activity of phenylalanine hydroxylase [58]. Tyrosine affects energy metabolism and immunity. Tryptophan can regulate oxidative stress, immune responses, and inflammation [40]. Consequently, the influence of these metabolic pathways may lead to impaired cellular immune defense function.

It is noteworthy that the TAP-treated group displayed a substantial increase in cystathionine content, suggesting an influence on the cysteine and methionine metabolic pathways (Figure 5C). Cystathionine is produced from the metabolism of methionine and is further hydrolyzed to generate cysteine, which is involved in the synthesis of GSH. This pathway is critical for cellular redox homeostasis [59]. In the research conducted by Chen et al. [60], it was observed that plasma cystathionine concentrations were notably higher in colorectal cancer patients as compared to individuals who were in good health. Therefore, the significant increase in cystathionine content may represent an imbalance in the redox state of A549 cells caused by TAP treatment.

#### 4.2.4. Analysis of Metabolic Pathways Affected by PEPA

The concentrations of metabolites affected by PEPA were all downregulated in cells (Table S6). Most of them are associated with key metabolic pathways: leucine, isoleucine, and valine are BCAAs related to blood glucose regulation and energy metabolism; glycine, serine, and threonine are essential precursors for protein synthesis; pyroglutamic acid, glycine, and inositol are related to the oxidative stress state of cells; and alanine, glucose, and lactic acid are related to energy metabolism. Referring to the correlation analysis of the other three flame retardants in this text, the downregulation of the above substances indicated that PEPA induced an imbalance in the redox state in A549 cells and affected the metabolism of amino acids and energy. It is worth noting that, although PEPA exhibited minimal cytotoxicity at high concentrations, its effects on cell metabolism were nonnegligible at low concentrations. The octanol–water partition coefficient (log *K*_ow_) is a valuable parameter to describe the quantitative structure–activity relationships of compounds in toxicology [61]. Therefore, from this perspective, the negative value for log *K*_ow_ of PEPA indicates that the substance did not easily enter cells and bioaccumulate, so it showed a lower cytotoxicity. However, from a metabolic standpoint, PEPA presents a certain degree of health risk.

## 5. Conclusions

In this study, we analyzed metabolite alterations in A549 cells using the KEGG and SMPDB databases combined with bioinformatics analysis methods. Our studies suggest that exposure to CDP, TAP, and PEPA may potentially lead to oxidative stress at the cellular level and disrupt amino acid and energy metabolism. RDP exposure has been shown to have a strong correlation with carbohydrate metabolism. These findings will broaden the research scope of OPFRs and help us gain a better understanding of the toxicity mechanism of eOPFRs in humans.

## Figures and Tables

**Table 1:** Chemical information.

Abbr.	CAS	MF	Log *K*_ow_	BCFs	Structure
CDP	26444-49-5	C_19_H_17_O_4_P	5.25	1019.28	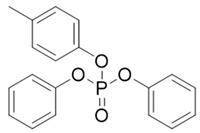
RDP	57583-54-7	C_30_H_24_O_8_P_2_	7.41	15,546.47	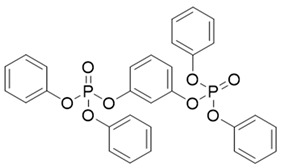
TAP	1623-19-4	C_9_H_15_O_4_P	1.94	17.85	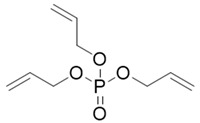
PEPA	5301-78-0	C_5_H_9_O_5_P	−1.76	1.00	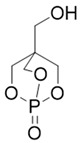

Note: Abbr. = abbreviation; CAS = chemical abstract services registry number; MF = Molecular formula; Log *K*_ow_ (octanol–water partition coefficient) = Log *K*_ow_ values; BCFs = bioconcentration factors; The data employed were retrieved from databases [5,17].

## Data Availability

The data presented in this study are available upon request from the **c**orresponding author.

## Supplementary Materials

The following supporting information can be downloaded at: https://www.mdpi.com/article/10.3390/toxics12060384/s1, Table S1: Summary of different metabolites in intracellular extracts of A549 cells; Table S2: Effects of different concentrations of eOPFRs on A549 cell activity for 48 h; Table S3: Altered metabolites of A549 cells after CDP exposure; Table S4: Altered metabolites of A549 cells after RDP exposure; Table S5: Altered metabolites of A549 cells after TAP exposure; Table S6: Altered metabolites of A549 cells after PEPA exposure; Figure S1: Summary plot of chromatograms for control and eOPFRs-treated groups.
