# Supplementary material for: GC/MS-Based Metabolomic Analysis of A549 Cells Exposed to Emerging Organophosphate Flame Retardants
Source: Toxics. 2024 May 24;12(6):384. doi: 10.3390/toxics12060384 (PMC11207991; doi:10.3390/toxics12060384)
Supplement: Supplementary file 1 [file toxics-12-00384-s001.zip › toxics-2981914-supplementary.pdf]

toxics

MDPI

Supplementary Material

Table S1. Summary of different metabolites in intracellular extracts of A549 cells.

No.	Metabolites	RT (min)	Characteristic Ion
1	Cystathionine	5.68	56
2	Lactic acid[a]	6.67	117
3	Glycol	6.82	191
4	2,3-Butanediol	7.06	129
5	Alanine[a]	7.48	116
6	Methylmalonic acid	7.55	131
7	Glycine[a]	7.78 10.89	102 174
8	Oxalic acid	8.16	147
9	2-Aminobutyric acid	8.73	130
10	Valine[a]	9.48	144
11	Urea	9.76	189
12	Leucine[a]	10.36	158
13	Phosphoric acid[a]	10.44	299
14	Isoleucine[a]	10.69	158
15	Serine[a]	11.70	204
16	Threonine[a]	12.08	117
17	2-Aminomalonic acid	13.20	320
18	Malic acid[a]	13.40	233
19	Erythritol	13.72	307
20	Pyroglutamic acid[a]	13.82	156
21	Proline[a]	14.57	142
22	Glutamic acid[a]	14.98	246
23	Phenylalanine[a]	15.09	218
24	P-hydroxyphenylacetic acid	15.29	323
25	2,6-Dihydroxybenzoic acid	15.36	355
26	Ribose[a]	15.80	103
27	4-Methylcatechol	16.70	253
28	Phloroglucinol	17.77	342
29	Fructose[a]	17.92 18.02	307
30	Glucose[a]	18.19 18.38	205
31	Mannitol[a]	18.60	319
32	Gluconic acid	18.72	217
33	Hexapyranose	18.94	204
34	Palmitic acid[a]	19.40	313
35	Inositol[a]	20.05	305
36	Stearic acid[a]	21.10	341
37	Oleic acid[a]	20.88	339
38	3-Phosphoglyceric acid	21.93	315
39	Glucose-6-phosphate	22.05	387
40	Uridine[a]	23.01	217
41	Glyceryl Monopalmitate	23.94	371
42	Glyceryl Monostearate	25.35	399

Note: RT, retention time ofmetabolites in chromatography.
2-Aminobutyric acid is the internal standard.
"a" means the metabolite was identified by standard compound.

Table S2. Effects of different concentrations of eOPFRs on A549 cell activity for 48 h.

Compound Concentration (μM)	Cell Viability (%) (n≥3, mean ±SEM)				P value
	CDP	RDP	TAP	PEPA	

0	100.70±1.20	100.30±0.33	100.3±1.45	99.67±0.88	>0.05
0.1	96.33±1.76	94.67±1.45	95.33±2.33	96.00±1.73	>0.05
1	93.67±2.40	94.13±1.62	94.00±2.65	94.00±2.08	>0.05
10	91.17±1.97	92.93±1.07	92.00±3.06	92.67±1.45	>0.05
100	86.00±0.58	88.67±0.88	85.67±2.03	88.33±1.20	<0.05
1000	34.67±1.45	71.57±3.90	46.00±2.08	79.00±1.53	<0.01

Table S3. Altered metabolites of A549 cells after CDP exposure.

HMDB	Metabolites	VIP	T-test(P)	Change	Fold
HMDB0000687	Leucine	1.264	0.026	↓	4.766
HMDB0000123	Glycine	1.249	0.008	↓	2.431
HMDB0000167	Threonine	1.210	0.02	↓	2.854
HMDB0000883	Valine	1.196	0.024	↓	2.894
HMDB0000148	Glutamic acid	1.191	0.027	↑	2.876
HMDB0001147	2-Aminomalonic acid	1.188	0.025	↓	3.089
HMDB0000625	Gluconic acid	1.181	0.028	↓	3.792
HMDB0000172	Isoleucine	1.162	0.035	↓	3.130
HMDB0000873	4-Methylcatechol	1.161	0.017	↑	1.827
HMDB0000162	Proline	1.159	0.03	↓	3.003
HMDB0062263	Serine	1.150	0.038	↓	3.946
HMDB0000161	Alanine	1.150	0.021	↓	2.669
HMDB0000267	Pyroglutamic acid	1.141	0.043	↓	4.114
HMDB0000122	Glucose	1.125	0.048	↓	5.270
HMDB0000190	Lactic acid	1.077	0.049	↓	2.296

Table S4. Altered metabolites of A549 cells after RDP exposure.

HMDB	Metabolites	VIP	T-test(P)	Change	Fold
HMDB0000883	Valine	1.307	0	↓	2.582
HMDB0000660	Fructose	1.279	0.001	↓	5.338
HMDB0000211	Inositol	1.279	0.001	↓	7.201
HMDB0000172	Isoleucine	1.269	0	↓	2.126
HMDB0000765	Mannitol	1.269	0.001	↓	3.156
HMDB0000122	Glucose	1.261	0.002	↓	10.724
HMDB0002994	Erythritol	1.236	0.001	↓	2.300
HMDB0000625	Gluconic acid	1.232	0	↓	5.336
HMDB0000687	Leucine	1.230	0	↓	1.909
HMDB0000267	Pyroglutamic acid	1.215	0.001	↓	4.369
HMDB0003345	Hexapyranose	1.203	0.001	↓	7.151
HMDB0000162	Proline	1.169	0.003	↓	2.293
HMDB0000744	Malic acid	1.158	0.002	↓	4.366
HMDB0000207	Oleic acid	1.116	0.006	↑	1.865
HMDB0003156	2,3-Butanediol	1.101	0.023	↑	1.816
HMDB0001147	2-Aminomalonic acid	1.065	0.023	↓	2.389
HMDB0000020	p-Hydroxyphenylacetic acid	1.058	0.014	↓	1.716
HMDB0000167	Threonine	1.052	0.025	↓	1.845

Table S5. Altered metabolites of A549 cells after TAP exposure.

HMDB	Metabolites	VIP	T-test(P)	Change	Fold
HMDB0000122	Glucose	1.435	0	↓	4.606
HMDB0000172	Isoleucine	1.407	0	↓	2.429
HMDB0000660	Fructose	1.405	0	↑	2.662
HMDB0000883	Valine	1.397	0	↓	2.332
HMDB0000687	Leucine	1.391	0	↓	2.322
HMDB0000625	Gluconic acid	1.387	0	↓	3.115
HMDB0003345	Hexapyranose	1.374	0.001	↓	6.402
HMDB0000159	Phenylalanine	1.337	0.001	↓	2.987
HMDB0000167	Threonine	1.335	0	↓	1.893

HMDB0000099	Cystathionine	1.296	0.001	↑	2.769
HMDB0000267	Pyroglutamic acid	1.270	0.002	↓	2.987
HMDB0000162	Proline	1.254	0.003	↑	2.276
HMDB0001881	Glycol	1.196	0.005	↑	2.099
HMDB0000873	4-Methylcatechol	1.166	0.018	↑	1.479
HMDB0002994	Erythritol	1.078	0.018	↑	1.869

Table S6. Altered metabolites of A549 cells after PEPA exposure.

HMDB	Metabolites	VIP	T-test(P)	Change	Fold
HMDB0000167	Threonine	1.401	0	↓	1.942
HMDB0000687	Leucine	1.376	0	↓	2.223
HMDB0000883	Valine	1.368	0	↓	2.159
HMDB0000172	Isoleucine	1.357	0	↓	2.192
HMDB0000161	Alanine	1.352	0	↓	1.860
HMDB0003345	Hexapyranose	1.344	0	↓	3.820
HMDB0000122	Glucose	1.312	0.001	↓	3.936
HMDB0000625	Gluconic acid	1.306	0.001	↓	2.631
HMDB0000123	Glycine	1.284	0.001	↓	1.525
HMDB0000211	Inositol	1.226	0.012	↓	1.252
HMDB0062263	Serine	1.207	0.007	↓	2.547
HMDB0000267	Pyroglutamic acid	1.184	0.017	↓	1.613
HMDB0000190	Lactic acid	1.161	0.015	↓	3.337
HMDB0031075	Glyceryl Monostearate	1.047	0.037	↓	1.298

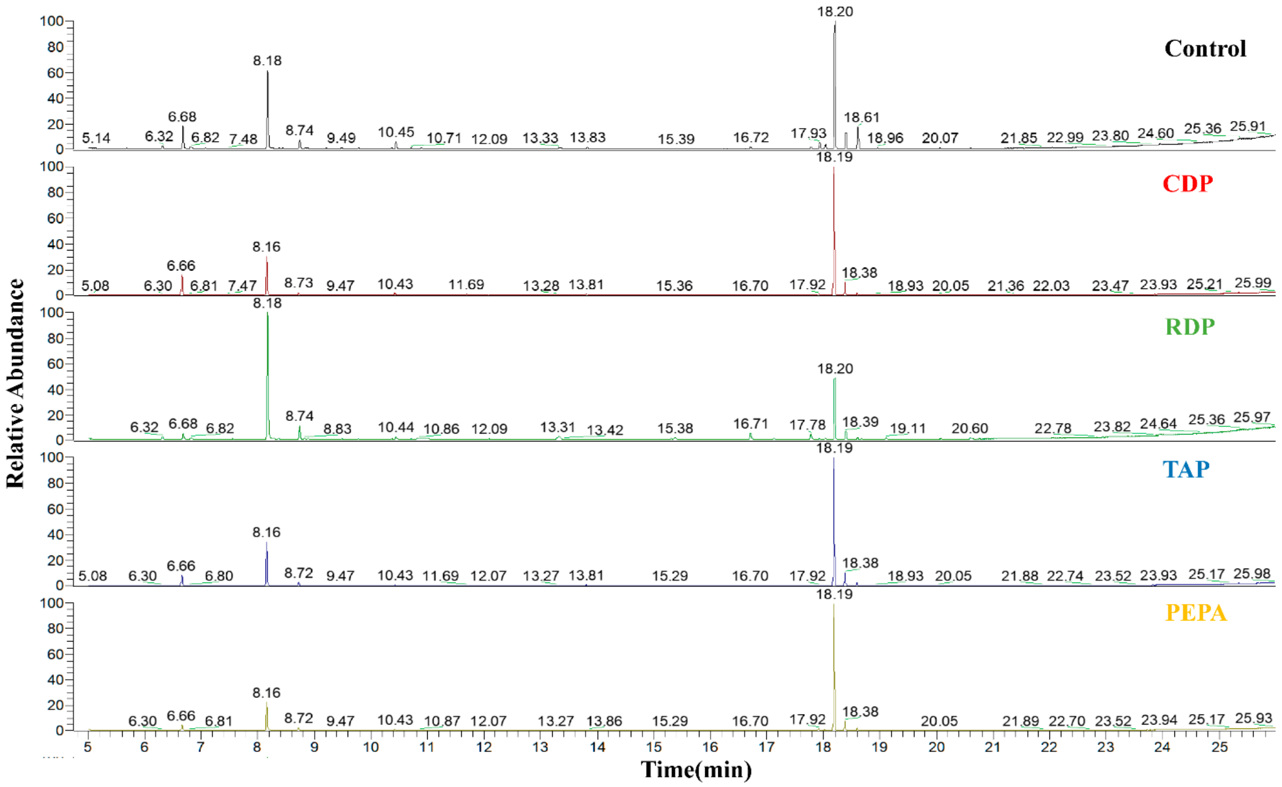

Figure S1. Summary plot of chromatograms for control and eOPFRs-treated groups.

GC/MS analysis

GC/MS measurements were conducted within the Trace ISQ™GC/MS system (Thermo Fisher Scientificial, USA), which was coupled to the XCalibur data processing system. Rxi-5ms capillary column (5% diphenyl/95% dimethylpolysiloxane, 30 m × 0.25 mm × 0.35 μm; Restek, USA) for chromatographic separation. The oven temperature program started at 80°C and Maintain 5 minutes, then heat to 300°C at 10°C/minutes and hold

for 5 minutes. The carrier gas used was helium (99.999%) at a constant flow rate of1 mL/min. The injection volume was 0.5μL and the split ratio was 50:1. The temperatures of transfer line, ion source and injector were all kept at 250 ◦C. Ionization was achieved using a 70 electronvolt electron beam. Mass spectra were recorded in full-scan mode, after 5 minutes of solvent delay, in the range of m/z 50-650.
